# Aberrant splicing and drug resistance in AML

**DOI:** 10.1186/s13045-016-0315-9

**Published:** 2016-09-10

**Authors:** Rosalia de Necochea-Campion, Geoffrey P. Shouse, Qi Zhou, Saied Mirshahidi, Chien-Shing Chen

**Affiliations:** 1 Biospecimen Laboratory, Loma Linda University Cancer Center, Loma Linda University School of Medicine, Loma Linda, CA 92354 USA; 2 Division of Hematology/Oncology, Loma Linda University School of Medicine, 11175 Campus Street, Chan Shun Pavilion 11015, Loma Linda, CA 92354 USA

**Keywords:** Splice factor, Chemoresistance, Mutation, Target, Clonal evolution

## Abstract

The advent of next-generation sequencing technologies has unveiled a new window into the heterogeneity of acute myeloid leukemia (AML). In particular, recurrent mutations in spliceosome machinery and genome-wide aberrant splicing events have been recognized as a prominent component of this disease. This review will focus on how these factors influence drug resistance through altered splicing of tumor suppressor and oncogenes and dysregulation of the apoptotic signaling network. A better understanding of these factors in disease progression is necessary to design appropriate therapeutic strategies recognizing specific alternatively spliced or mutated oncogenic targets.

## Background

Chemoresistance is a critical challenge in the management of acute myeloid leukemia (AML) disease. While many patients respond well to induction therapy, the majority relapse due to partial or developed clinical resistance [1]. In general, AML prognosis is broadly stratified based on patient age. Patients 60 years old or younger have a 35–40 % chance of achieving a cure while those older than 60 have only a 5–15 % chance [2]. Yet, AML prevalence is much more common among the elderly with a median age of diagnosis of 69 years [3]. Older AML patients do not tolerate aggressive treatments as well as younger patients and tend to have more resistant clonal variants of the disease making it difficult to achieve a cure [4]. The potential for development of splice variant specific targeting therapies may help reduce treatment toxicity in this older patient population.

**Fig. 1:**
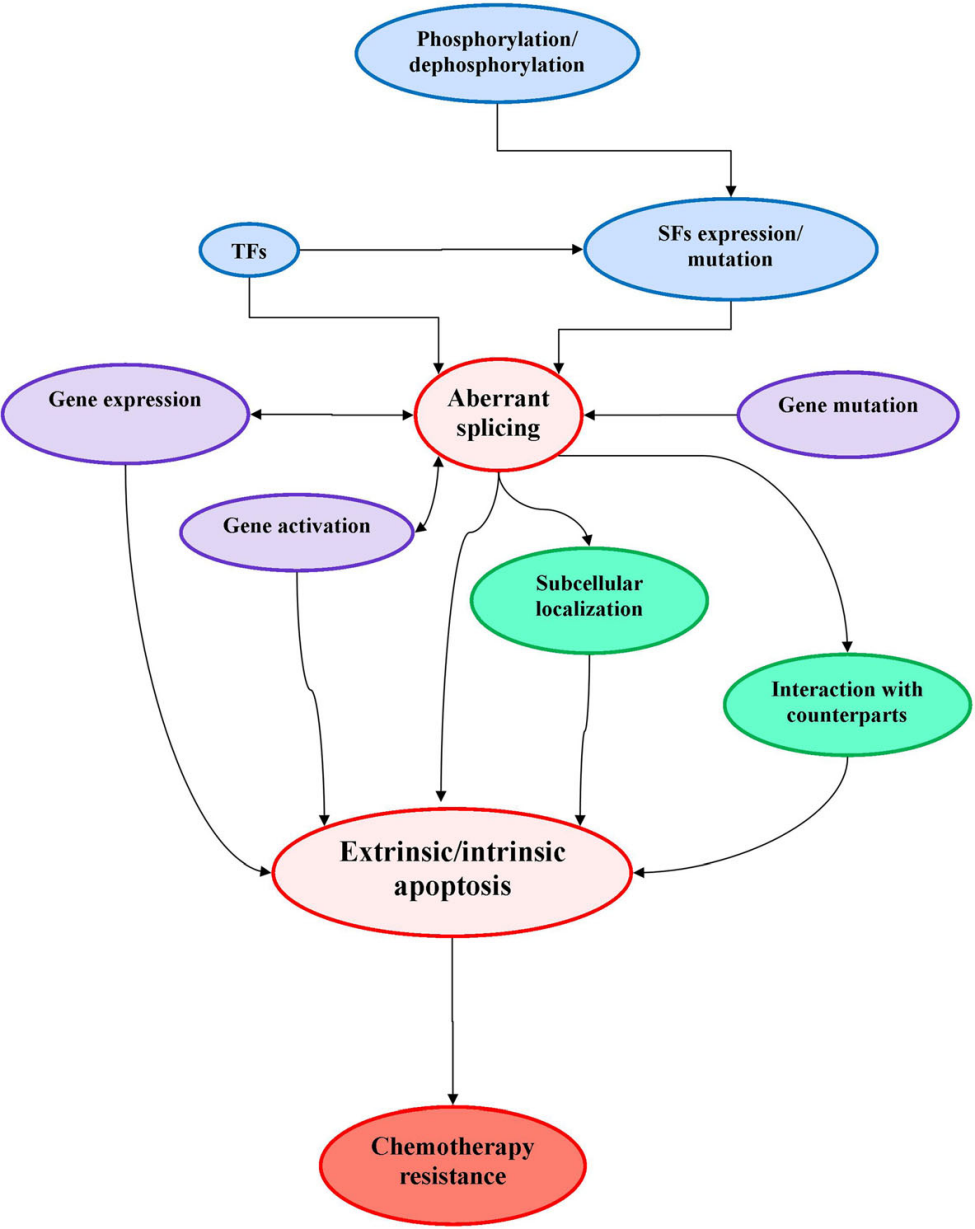
Schematic diagram of factors influencing aberrant splicing and drug resistance in AML. *TF* transcription factor, *SF* splice factor

The role of gene splicing abnormalities in AML disease progression and drug resistance has gained attention as several recent studies have highlighted recurrent splice factor mutations as important drivers of hematological malignancies [5]. Splice factors are proteins that together with at least five small nuclear ribonucleic acids (RNAs) form part of a dynamic complex called the spliceosome which splices a nascent RNA transcript at precise locations to form a mature messenger RNA (mRNA) sequence that can code for a functional protein [6]. Splicing is a highly regulated process that can involve a coordinated action among more than 200 molecules to favor or repress splicing at specific target sites [1]. There are two well-known protein families of RNA-binding splice factors: the serine-rich (SR) proteins that typically promote exon inclusion and the heterogenous nuclear ribonucleoproteins (hnRNP) that generally promote exon skipping [7]. Alternative splicing is an important part of normal hematopoiesis and is necessary for cellular differentiation and rapid responses to external stimuli [8]. However, imbalances in the splicing machinery can trigger abnormal programs associated with malignancies such as myelodysplastic syndromes (MDS) and AML [9]. As shown in Fig. 1, dysregulation of splicing mechanisms can affect apoptotic susceptibility in AML and lead to drug resistance.

## Recurrent splice factor mutations in myeloid neoplasms

Next-generation sequencing technologies have revealed a striking number of myeloid neoplasms harboring splice factor mutations that alter global splicing events [9]. More than half of patients with MDS have mutations within functional components of the spliceosome that are considered important disease founding events [10]. The most common recurrent mutations among patients with MDS are found among the serine-rich SF3B1, SRSF2, and U2AF1 splice factors [11]. Approximately, 19–28 % of MDS patients have SF3B1 mutations [12], 12.4 % have SRSF2 mutations [13], and 6.3 % have U2AF1 mutations [14]. Splice factor mutations have genome-wide effects that alter splicing patterns for hundreds of genes. In MDS patients harboring SF3B1 mutations, 526 genes were found to be differentially expressed and 2022 genes were alternatively spliced when compared with CD34+ cells from MDS patients without any splicing mutations [15]. In K562 and TF1 myeloid cell lines with SF3B1, knockdown 1419 genes were differentially expressed and 384 genes were differentially spliced [15]. In K562 cells expressing mutant versions of the U2AF1 splice factor, 259–922 genes were differentially spliced depending on the type of mutation [16]. Intriguingly, only 17 % of the alternate splicing events detected in K562 cells with U2AF1 mutants overlapped with those detected in samples from AML patients harboring the same point mutations, suggesting that context-specific expression of other factors also strongly influences this outcome [16]. In an MDS cell line expressing a mutant version of the SRSF2 splice factor, 487 genes were found to be differentially spliced [17]. In general, SF3B1, SRSF2, and U2AF1 splice factor mutations tend to promote exon skipping during the splicing process as their ability to recognize specific RNA 3′ splice site sequences is usually affected by the mutation [5].

The SF3B1, SRSF2, and U2AF1 splice factor mutations have garnered substantial attention due to their frequent, though not indispensable, presence in myeloid neoplasms. However, many other rare splice factor mutations such as SF3A1 or PRPF40B can also exert widespread influence on alternative splicing of target RNA sequences [9]. It has been shown that spliceosome mutations tend to occur in a mutually exclusive, rather than synergistic, manner [18], suggesting a selective mechanism regulating the production of alternate protein isoforms involved in cell function and disease progression. However, not all splice factor mutations have similar adverse associations with disease development and patient prognosis as some are linked to favorable clinical outcomes [11, 12].

## Splicing in AML

Intriguingly, splice factor mutations are less common in AML than MDS, despite AML sometimes arising from an important transformative event in MDS progression that occurs in about one third of MDS patients [19]. In general, the prevalence of more common splice factor mutations in AML is approximately 4 % for SF3B1, 4.9 % for SRSF2, and 6.4 % forU2AF1 [5]. In MDS patients, SF3B1 mutations are associated with better clinical outcomes and reduced risk of AML development [12]. In contrast, SRSF2 mutations predict shorter survival outcomes and greater risk of AML progression [13]. U2AF1 mutations carry the greatest risk of progression to AML [19] and are associated with a lack of remission and short survival outcomes in AML patients [20]. Poor response to therapy and adverse patient outcomes suggest that these aberrant splicing events strongly influence tumor cell survival.

Accordingly, recent studies have demonstrated that alternative splicing events may be a fundamental aspect of AML disease biology. A genome-wide analysis of aberrant splicing patterns in AML patients showed that approximately one third of genes are differentially spliced compared with CD34+ cells obtained from normal controls [21]. In two study cohorts, totaling more than 200 AML patients, 135–786 recurrently spliced genes were identified in each patient sample [21]. Approximately 76–80 % of these splicing changes could be mapped to the translated transcript regions likely altering some aspects of protein function, while changes to the untranslated region could affect transcript stability or translation efficiency [21]. About half of the splice variants that were identified had not been previously reported [21], suggesting a disease-specific etiology. In multiple patient samples, presence and abundance of some splice variants could only be observed at diagnosis, disappeared during remission, and then strongly re-expressed during relapse [21].

## Chemoresistance and apoptotic signaling

Chemoresistance in AML is often linked to defects in the apoptotic signaling network. There are two well-characterized pathways of apoptotic programmed cell death that are distinguished by induction mechanisms and specific molecular signaling events. The extrinsic apoptotic pathway requires activation of a death receptor on the cell surface which initiates CASP8 cleavage of downstream executioner caspases [22]. In contrast, the intrinsic apoptotic pathway requires intracellular stress signals that induce mitochondrial membrane permeabilization to activate CASP9 which then cleaves and activates the downstream executioner caspases [23]. Mitochondrial membrane susceptibility to permeabilization is primarily controlled by the B cell lymphoma 2 (BCL-2) family of proteins which has both pro- and anti-apoptotic members that cumulatively regulate mitochondrial membrane integrity [24]. Multiple studies have shown that basal expression levels of BCL-2 proteins can help predict response to chemotherapeutic agents in AML [25, 26]. In particular, overexpression of anti-apoptotic members of this protein family is sufficient to establish tumor cell resistance to multiple types of cytotoxic stimuli, although cell dependence on a specific protein mechanism for survival varies by treatment [25]. Similarly, upregulation of inhibitor of apoptosis (IAP) protein members, which block apoptosis by inhibiting caspase activity, is associated with chemoresistance in multiple types of cancers including AML [27].

## SRSF1 splice factor

SRSF1 is an oncogenic splice factor that regulates splicing of several proteins in the apoptotic pathway. SRSF1 overexpression was able to induce tumor formation in epithelial cells and inhibit apoptosis in breast cancer cells [28]. SRSF1 promotes alternative splicing of Bcl-2-like protein 11 (BIM) producing isoforms lacking pro-apoptotic functions [28]. Furthermore, SRSF1 expression is linked to mTORC1 activation [29] which is a signaling pathway associated with AML progression and clonal selection during re-propagation from minimal residual disease [30]. Downregulation of SRSF1 in cervical carcinoma cells promoted pro-apoptotic splicing of *MCL-1*_*S*_, *BCL-X*_*S*_, *CASP9*, and *CASP2* variants [31]. Perhaps, this is partially explained by a shift in the functional balance with RBM4, a tumor suppressor splice factor that competitively binds with SRSF1 to specific regulatory sequence elements found in splicing locations of pre-mRNA [32]. In NSCLC cells, overexpression of RBM4 was found to neutralize SRSF1 to inhibit mTOR activation and induce apoptosis by shifting *BCL-X* splicing to favor the pro-apoptotic *BCL-X*_*s*_ isoform [32].

## FLT3 pathway

One of the most common deregulated signaling mechanisms in AML is that of FLT3, a tyrosine kinase receptor that is frequently mutated and constitutively active in about 25 % of AML patients [33]. Furthermore, *FLT3* is one of the most highly mis-spliced genes in AML with aberrant splicing of the transcript primarily altering an extracellular region of this receptor [34]. These alterations are likely to be associated with AML pathogenesis as certain *FLT3* splice variants are strongly expressed during diagnosis and relapse, but not detected during remission, nor in other types of hematologic malignancies [34]. Intriguingly, the production of *FLT3* splice variants is not associated with the presence of any specific splice factor mutations [34] suggesting changes in expression of regulatory spliceosome elements may promote these events. *FLT3* splice variants encode functional proteins and affect critical downstream signaling targets (AKT, STAT, ERK) which are highly activated in AML cells producing aberrant *FLT3* transcripts, even in the absence of an inducing FLT3 ligand [34]. Variable activation of downstream signals may help to explain why only about 30 % of AML patients with activating FLT3-ITD mutations become resistant to treatment with FLT3 inhibitors [33]. Previous research in our lab demonstrated that AML cells with FLT3-ITD mutations that acquired resistance to one type of FLT3 inhibitor were also resistant to other structurally unrelated FLT3 inhibitors due to upregulation of a downstream STAT3-survivin pro-survival pathway [35].

## Survivin splice variants

Even when anti-apoptotic splice variants have a similar cellular function, drug response can be altered by the ratio of variants. Survivin is a protein that can inhibit apoptosis by stabilizing other anti-apoptotic proteins such as X-linked inhibitor of apoptosis protein (XIAP) and sequestering pro-apoptotic proteins such as Smac/Diablo [36, 37]. At least five alternative splice variants of survivin have been reported that also conserve the XIAP and Smac/Diablo protein binding regions but have distinct levels of association with chemoresistance and other important clinico-pathological features in cancer [38]. Presumably, any type of structural change can alter the affinity and functional outcome of survivin interactions. In addition, survivin splice variants have distinct subcellular compartmentalization patterns that can shift upon exposure to chemotherapy [39]. In HeLa cells treated with etoposide, a mitochondrial accumulation of survivin splice variant DeltaEx3 inhibited apoptosis by preventing release of Smac/Diablo [39]. In AML patients, high expression of *survivin-2B* is significantly associated with risk of refractory disease and poor survival outcomes in both children [40] and adults [41].

## AKT activity and caspase-9 splicing

AKT activity has been linked to several aberrant splicing mechanisms that promote chemoresistance, primarily through modulation of *CASP9* splicing. There are two alternative splice variants of *CASP9* distinguished by the inclusion (*CASP9a*) or exclusion (*CASP9b*) of a four-exon cassette to create pro- or anti-apoptotic isoforms, respectively [42]. In non-small cell lung cancer (NSCLC), AKT phosphorylation of the splice factor srp30a (SRSF1) promotes splicing of the anti-apoptotic isoform, *CASP9b* [42]. One of the exons excluded from anti-apoptotic *CASP*9, exon 3, is found to differentially select through a competitive interaction between the splicing enhancer hnRNPU and repressor hnRNPL, which is influenced by AKT activity [43]. AKT phosphorylation of hnRNPL displaces hnRNPU at the exon 3 binding location of *CASP9*, effectively decreasing the ratio of *CASP 9a/b* and enhancing the tumorigenic potential of NSCLC cells [43]. Multiple studies, including recent research in our lab, have demonstrated that higher expression of AKT is associated with a drug-resistant phenotype in AML [44].

## WT1 and E2F1 transcription factors

Transcription factors can also have a critical regulatory role in the splicing process and development of chemoresistance. Wilms tumor 1 (WT1) is a zinc finger transcription factor of important prognostic significance in AML with four predominant splice isoforms, one of which represses transcription of the SRPK1 splice factor and alters VEGF splicing, promoting production of the anti-angiogenic VEGF-A_165_b isoform [45]. While WT1 was originally identified as a tumor suppressor of kidney cancer, its critical role in AML development in transgenic mice transduced with AML1-ETO [46], resistance to death stimuli [47], and importance as a prognostic marker of patient relapse and survival [48] demonstrate its functions as an oncogene in AML. WT1 has been shown to interact directly with the splice factors RBM4, U2AF2, and WTAP in an isoform-dependent manner, which can influence splicing outcomes of their target transcripts [45]. In fact, knockdown of WT1 expression in AML cell lines promoted alternative splicing events affecting approximately 1200 genes [47]. E2F transcription factor 1 (E2F1) is another transcription factor that influences apoptotic susceptibility of tumor cells by regulating alternative splicing of apoptotic genes [49]. E2F1 has a role in induction of apoptosis caused by DNA damaging agents, by upregulating expression of the splicing factor SC35 (U2AF1) which promotes selection of the pro-apoptotic variants of FLICE-like inhibitory protein (c-FLIP), CASP8, CASP9, and BCL-X [50]. Yet, while E2F1 may be an important tumor suppressor, when this transcription factor interacts with cofactors commonly expressed by tumor cells with defective cell death signaling pathways, it participates in feedback loops that can contribute to tumor aggressiveness and chemoresistance [51]. It was shown that knockdown of E2F1 resulted in approximately a 50 % decrease of cell viability in chemoresistant lung adenocarcinoma cells exposed to multiple therapeutics [52]. Therefore, E2F1 molecular targeting may be a beneficial therapeutic strategy for certain aggressive tumor phenotypes.

## Extrinsic apoptotic signals

Changes in expression of some splice factors such as SPF45 can modify extrinsic apoptotic signaling mechanisms in ways that lead to increased drug resistance [53]. For example, overexpression of SPF45 in HeLa cells resulted in up to a 21-fold increased resistance to multiple chemotherapeutics (carboplatin, vinorelbine, doxorubicin, etoposide, mitoxantrone, and vincristine), while SPF45 knockdown sensitized these tumor cells to some treatment conditions [54]. One of the few identified splicing targets of SPF45 is the *Fas* death receptor transcript for which SPF45 promotes an exon skipping event to produce a soluble anti-apoptotic protein isoform lacking the transmembrane domain [53]. Nearly 200 genes have been identified in mammalian cells that can modulate splicing of *Fas* transcripts [55], and AML cells exposed to different chemotherapeutics dysregulate *Fas* splicing patterns [56]. Elevated levels of soluble Fas in AML patient sera can inhibit apoptosis of leukemic blasts by sequestering the Fas ligand and have been associated with patient relapse and resistance to therapy [57]. Fas death receptor signaling can also be inhibited by alternative splicing of CASP8 and production of an anti-apoptotic variant, CASP8_L_ [58]. CASP8_L_ is upregulated in hematopoietic progenitor cells and minimally differentiated AML [59], characterized as a particularly chemoresistant AML subtype [60]. A second group of extensively studied death receptors with adverse prognostic associations in AML are the TNF-related apoptosis-inducing ligand (TRAIL) death receptors [61]. The IG20 gene, with a role in transduction of TRAIL death receptor signals, produces four predominant splice isoforms with contrary effects on apoptosis due to an ability to interfere with or promote CASP8 activation [62]. Anti-apoptotic IG20 splice variants (DENN) are expressed at high levels in different types of leukemia cells causing TRAIL associated apoptotic resistance [63].

## c-FLIP

c-FLIP is another protein associated with chemoresistance and cell survival that can interfere with activation of the extrinsic apoptotic signaling pathway [64]. It is predominantly expressed as two functional splice isoforms (c-FLIP_L_ and c-FLIP_S_) which block activation of CASP8 through heterodimerization at different steps in the death induction signaling pathway [65] but can also have dual roles depending on the physiological context. The longer isoform (c-FLIP_L_) inhibits apoptosis when expressed at high concentrations but enhances CASP8 activation and promotes apoptosis when expression levels are low [66]. In AML, c-FLIP splicing patterns can have an important prognostic value as the high expression levels of c-FLIP_L_, but not c-FLIP_s_, are associated with significantly shorter survival outcomes [67]. However, both c-FLIP isoforms may be important therapeutic targets in AML since they activate cytoprotective pathways involved in disease progression [64], and dual c-FLIP downregulation can induce or sensitize AML cells to apoptosis [67].

## Extrinsic and intrinsic apoptotic link

BH3 interacting-domain death agonist (BID) is a key player in the apoptotic signaling network since it has the ability to link the extrinsic to the intrinsic apoptotic pathways [22]. When CASP8 is activated by extrinsic death receptor signaling, it can activate BID which then translocates to the mitochondria and interacts with other pro-apoptotic BCL-2 members to promote intrinsic apoptosis [68]. However, alternative splicing events are known to produce at least four functional BID isoforms with distinct subcellular localization patterns and conflicting apoptotic roles [69]. Pro-apoptotic BID is essential for normal hematopoiesis, and BID-deficient mice spontaneously develop fatal myeloid neoplasms indicating that BID is necessary to suppress leukemogenesis [70]. The presence of pro-apoptotic BID can also determine tumor cell susceptibility to extrinsic apoptotic stimuli [71], suggesting that both quantity and ratio of BID splice variants can strongly influence AML development and chemoresistance.

## Anti-apoptotic BCL-2 proteins

Aberrant splicing of several BCL-2 family members is known to promote drug resistance in AML. In this family, *MCL-1*, *BCL-X*, and *BID* have splice variants coding for protein isoforms with contrary apoptotic functions [68]. The predominantly expressed MCL-1 and BCL-X proteins are the longer anti-apoptotic isoforms (MCL-1_L_ and BCL-XL) that can bind pro-apoptotic BAK preventing its oligerimerization and permeabilization of the mitochondrial membrane [72]. MCL-1_L_ can also sequester pro-apoptotic proteins BIM and BID and, among multiple anti-apoptotic BCL-2 members, was found to be a critical regulator of AML cell survival [72]. *MCL-1* splicing is partially regulated by SF3B1, and inhibition of SF3B1 reversed the dominant splice isoform from anti-apoptotic *MCL-1*_*L*_ to pro-apoptotic *MCL-1*_*S*_ in NSCLC [73] and cervical carcinoma cell models [31]. Our research group recently demonstrated that exposure to YM155 significantly upregulated expression of *MCL-1*_*S*_ in AML cells sensitive to this treatment [44], although a splice factor mechanism modulating this event was not identified. However, other researchers have demonstrated that some drugs that block tumor growth and induce cell cycle arrest promote production of pro-apoptotic *MCL-1*_*S*_ and *BCL-X*_*S*_ variants through downregulation of the AS/SF2 (SRSF1) splice factor [31].

## Pro-apoptotic BCL-2

Alternative splicing of BH3-only pro-apoptotic BCL-2 proteins found in the intrinsic apoptotic signaling pathway can also contribute to a chemoresistant phenotype in AML. Among these BCL-2 members, only BIM and p53 upregulated modulator of apoptosis (PUMA) have a strong affinity to all anti-apoptotic BCL-2 proteins (BCL-2, BCL-XL, BCL-W, MCL-1, and A1), while BAD, BIK, HRK, and NOXA selectively heterodimerize with a limited number of anti-apoptotic members [74]. BIM has multiple alternative splice isoforms having distinct pro-apoptotic mechanisms due to altered protein heterodimerization abilities [75] and mitochondrial localization patterns [76]. In one study, inhibition of any of the three major *BIM* isoforms resulted in different levels of resistance to glucocorticoid treatment in leukemia cells [77]. Comparably, an intronic deletion in the *BIM* gene was found to promote production of an anti-apoptotic BH3-deficient splice isoform which was associated with resistance to tyrosine kinase inhibitor treatment in leukemia cell lines [78]. Similarly, NOXA also has at least two splice variants lacking the BH3 region which eliminates their ability to bind to other BCL-2 family proteins and results in a loss of pro-apoptotic activity [79].

## Other apoptotic regulators

Alternative splicing of many other proteins with regulatory roles in the apoptotic signaling network can enhance pro- or anti-apoptotic characteristics and influence chemoresistance. For example, a pro-apoptotic Smac3 splice variant of Smac/Diablo was found to accelerate XIAP autoubiquitination and destruction and enhanced apoptotic cell death in HeLa cells exposed to multiple chemotherapeutics [80]. XAF1 is a pro-apoptotic protein that also targets XIAP and can enhance tumor cell susceptibility to cisplatin [81] and radiotherapy [82]. While XAF1 expression in tumor cells is generally very low, quantities of alternatively spliced truncated isoforms lacking the XIAP interaction have been found to increase in some aggressive cancers and have been suggested as potential biomarkers of disease status [83, 84]. Frequently, when alternatively spliced isoforms have opposing functions, the ratio of one isoform to another is associated with a cancer treatment phenotype. For example, expression of anti-apoptotic neuronal apoptosis inhibitory protein (NAIP) relative to its less effective splice variant NAIP-DEx3 increases substantially in chemoresistant AML cell lines [85]. Intriguingly, one study performed with AML blasts from patients with relapsed chemoresistant AML, found the p75 splice variant of the lens epithelial-derived growth factor (LEDGF) protein to be the most consistently upregulated transcript in these cells [86]. LEDGF p75 is a stress oncoprotein that promotes chemoresistance but has an antagonistic splice isoform, LEDGF p52, that can promote apoptosis in tumor cells [87].

## Splice variant targeting therapies

A number of natural products derived from distinct species of bacteria have been found to target the SF3B component of the spliceosome and demonstrate potent antitumor activities [88]. One of the first to be identified was FR901464, a fermentation product from *Pseudomonas*, which has been used as a structural model for the synthesis of several stable chemical analogs of similar or greater potency [89]. In particular, meayamycin B, a soluble synthetic FR901464 derivative, can significantly inhibit tumor growth at low picomolar concentrations and demonstrates important potential for development as a novel therapy for AML treatment [90]. Meayamycin B inhibits the SF3B1 subunit and can shift alternative splicing of *MCL-1* to promote expression of the pro-apoptotic *MCL-1*_*s*_ isoform [73]. Although SF3B1 is among the most commonly mutated splice factors both in MDS and AML, these mutations are not located in the putative binding region of spliceosome inhibitors like meayamycin B, suggesting that they could be effective therapeutic options for patients with these mutations [91].

## Conclusions

In AML, generation of functional protein products with oncogenic qualities is largely determined by regulation of transcript expression levels and alternative splicing events [92]. Upregulation of pro-survival pathways, oncogenic transcription factors, intrinsic and extrinsic apoptotic signals, and production of altered death effector molecules are all important elements that can be altered by splicing events to establish a chemoresistant phenotype. During AML development, increasingly oncogenic transformations of a hematopoietic cell eventually result in proliferation of a founding clone and usually the presence of at least one subclone [93]. AML cells acquire additional mutations during disease progression in a process of clonal selection that can be strongly influenced by treatment such that a cytogenetically distinct clone is present at relapse [94]. During this process of clonal evolution, mutations causing dysregulation of alternative splicing events that confer tumor cell survival advantages can become important drivers of leukemogenesis and drug resistance [9]. AML patients with the highest rates of splice factor mutations share a common disease ontogeny characterized by a high incidence of treatment failure [95]. Although splice-typing is not yet a widespread method of AML classification or risk stratification, the potential to develop these analyses merits further evaluation. As understanding of splicing dysregulation in this disease grows, new therapeutic targets may become evident. Ultimately, treatment strategies that target key spliceosome elements such as SF3B1 or specific oncogenic splice variants in a manner that circumvents the resistance mechanism may prove to be valuable methods to eradicate these types of AML clones.

## Abbreviations

AML: Acute myeloid leukemia
MDS: Myelodysplastic syndromes
SR: Serine rich
hnRNP: Heterogenous nuclear ribonucleoproteins
RNA: Ribonucleic acid
SF3B1: Splicing factor 3b, subunit 1
SRSF2: Serine/arginine-rich splicing factor 2
U2AF1: U2 small nuclear RNA auxiliary factor 1
BCL-2: B cell lymphoma 2
IAP: Inhibitor of apoptosis
BIM: Bcl-2-like protein 11
SRSF1: Serine/arginine-rich splicing factor 1
mTORC1: Mammalian target of rapamycin complex 1
AKT: V-Akt murine thymoma viral oncogene homolog
STAT: Signal transducer and activator of transcription
ERK: Extracellular regulated kinase
FLT3: Fms-related tyrosine kinase 3
FLT3-ITD: FLT3 internal tandem duplication
Smac/Diablo: Second mitochondria-derived activator of caspase
WT1: Wilms tumor 1
E2F1: E2F transcription factor 1
VEGF: Vascular endothelial growth factor
AML1-ETO: AML1 and eight twenty-one protein fusion
RBM4: RNA-binding motif protein 4
U2AF2: U2 small nuclear RNA auxiliary factor 2
WTAP: Wilms tumor 1 associated protein
c-FLIP: FLICE-like inhibitory protein
BCL-X: Bcl-2-like protein 1
SPF45: Splicing factor 45
CASP8: Caspase-8
CASP9: Caspase-9
CASP2: Caspase-2
TRAIL: TNF-related apoptosis-inducing ligand
BID: BH3 interacting-domain death agonist
MCL-1: Myeloid cell leukemia 1
PUMA: p53 upregulated modulator of apoptosis
BAD: BCL2-associated agonist of cell death
BIK: Bcl2-interacting killer
BAK: Bcl-2 homologous antagonist/killer
HRK: Harakiri, Bcl-2 interacting protein
XIAP: X-linked inhibitor of apoptosis protein
XAF1: XIAP-associated factor 1
NAIP: Neuronal apoptosis inhibitory protein
LEDGF: Lens epithelial-derived growth factor
